# The Evolving Case Supporting Individualised Physiotherapy for Low Back Pain

**DOI:** 10.3390/jcm8091334

**Published:** 2019-08-28

**Authors:** Jon Ford, Andrew Hahne, Luke Surkitt, Alexander Chan, Matthew Richards

**Affiliations:** 1 School of Allied Health, La Trobe University, Kingsbury Drive, Bundoora 3086, Australia; 2 Advance Healthcare, 157 Scoresby Road, Boronia 3155, Australia

**Keywords:** low-back pain, physiotherapy, individualisation

## Abstract

Low-back pain (LBP) is one of the most burdensome health problems in the world. Guidelines recommend simple treatments such as advice that may result in suboptimal outcomes, particularly when applied to people with complex biopsychosocial barriers to recovery. Individualised physiotherapy has the potential of being more effective for people with LBP; however, there is limited evidence supporting this approach. A series of studies supporting the mechanisms underpinning and effectiveness of the Specific Treatment of Problems of the Spine (STOPS) approach to individualised physiotherapy have been published. The clinical and research implications of these findings are presented and discussed. Treatment based on the STOPS approach should also be considered as an approach to individualised physiotherapy in people with LBP.

## 1. Introduction

Low-back pain (LBP) is recognised as a common and costly problem in the Western world, with a global prevalence of 0.5 billion, the highest ranking cause of years lived with disability contributing 57·6 million years [1], and an increase in prevalence and disease burden of nearly 20% over the last 10 years [2]. People with LBP have historically been described as having a favourable natural history [3]; however, systematic reviews of primary care studies show that 28%–79% of people with acute LBP experience persistent or recurrent symptoms at 12 months [4,5]. Higher rates are supported by one large general population study which is likely to be a more accurate measure of persistency/recurrence than samples recruited from primary care settings [6].

Syntheses of clinical guidelines suggest international consensus in recommending initial exclusion of red flags and radiculopathy, and subsequent management of LBP as a “non-specific” condition on the basis that a nociceptive cause of symptoms cannot be identified [7,8,9]. Guideline-based treatment (Table 1) aims to minimise potential harm of treatments such as surgery or medication and maximise cost-effectiveness by utilising simple treatments such as advice [10,11]. However, the randomised controlled trials (RCTs) upon which guideline recommendations are based typically show small effect sizes of questionable clinical importance [8,9,12].

A potential reason for the limited effects demonstrated in RCTs on LBP is a false assumption that non-specific LBP is a homogeneous group. It has been postulated that multiple subgroups exist within the non-specific LBP population that are likely to respond differently to generic treatment [13]. In such circumstances, a false assumption of sample homogeneity in RCTs may lead to a treatment being inappropriately applied, resulting in either failure to respond or exacerbation of the condition. Based on this understanding, identifying valid subgroups for the purposes of an RCT has been described as a high research priority [14,15]. Meaningful subgroups enable treatment to be individualised to the patient presentation, potentially increasing the size of the effect [16]. An example of the value of individualised treatment is the management of inflammation in people with LBP. Guidelines suggest that non-steroidal anti-inflammatory drugs (NSAIDs) have small and short-term positive effects for LBP [8], yet these recommendations are based on RCTs selecting people with non-specific LBP. It is unlikely that every patient in this population has LBP with inflammatory processes as a contributing factor. It is, therefore, plausible that RCTs sampling populations with a greater likelihood of an inflammatory component to their LBP would show larger effects.

The argument for the importance of individualised treatment is further strengthened by considering the multi-dimensional nature of LBP. Clinical guidelines for LBP [9], the World Health Organisation’s International Classification of Functioning, Disability and Health [17] and internationally accepted standards on clinical reasoning [18] all emphasise multiple factors that are relevant for the management of LBP including the pathoanatomical (e.g., nociceptive source of symptoms), psychosocial (e.g., fear avoidance), neurophysiological (e.g., central sensitisation and neuropathic pain) and genetic dimensions. The complexity of LBP is also reflected in the wide range of subgrouping approaches reported in systematic and narrative reviews [13,19,20,21]. Given the multidimensional and complex nature of LBP, it is almost axiomatic that a “one size fits all” approach to treatment provision in RCTs is likely to yield suboptimal results [13].

Based on the scale of the LBP problem, the limited data on treatment effectiveness, and the potential value of individualised treatment, the aim of this paper was to overview the evidence on individualised physiotherapy, including a contextualised presentation and discussion of a series of studies on the Specific Treatment of Problems of the Spine (STOPS) approach.

## 2. The Evidence on Individualised Physiotherapy for Low-Back Pain

A search on the evidence supporting individualised physiotherapy for LBP was conducted on PubMed using the Boolean term OR for individ*, subgroup*, classif* AND back pain AND “review” in the title. Reference lists of the retrieved papers, as well as recent clinical guidelines [7,8,9], were also checked for relevant evidence. A total of 546 citations were identified from PubMed, with 12 being deemed relevant for this overview.

Individualising treatment for LBP has been identified as a high research priority by a series of international expert panels [14,15] and a methodological framework for future research suggested [19,22]. However, research investigating the large number of heterogenous approaches for individualising treatment are of variable methodological quality [20,23,24,25,26,27]. Individualising physiotherapy based on movement is recommended in a professional guideline [28] but is not supported by recent clinical trials [29]. The STarT Back approach to individualising physiotherapy has been extensively researched in different contexts [30,31,32,33,34,35,36,37,38,39,40,41,42], and is recommended in clinical guidelines based on cost effectiveness [8,9]. However, the STarT Back approach only confers small clinical effects on activity limitation, and no long-term effects on pain compared to usual care [33].

Given the limited evidence supporting attempts to develop effective individualised physiotherapy approaches for LBP, exploration of alternative methods has merit.

## 3. A Series of Studies Supporting Individualised Physiotherapy for Low-Back Pain

The STOPS trial was a randomised controlled trial (*n* = 300) published in 2016 that concluded individualised physiotherapy was more effective than guideline-based advice for early persistent LBP [43]. This trial was part of a series of studies that will be overviewed to inform a discussion on the STOPS approach to individualised physiotherapy for LBP.

### 3.1. Prognosis in Identifying Potential Targets for Individualised Physiotherapy

Identification of prognostic factors can improve clinical decision making, understanding of disease processes, definitions of risk groups, and prediction of clinical outcomes [44]. Prognostic factors can also assist in identifying treatment targets to improve the effectiveness of individualised treatment [45,46]. Exploring and identifying gaps in the prognostic literature for LBP has been recommended as a research priority [47].

Prognostic studies and systematic reviews on LBP commonly evaluate specific prognostic factors [47,48] such as psychosocial distress [49,50,51,52], clinical features [53,54,55,56] and physical activity [57]. We are unaware of any high-quality studies evaluating a comprehensive range of biomedical (including pathoanatomical), psychological and social prognostic factors using multivariate methods in a large sample of people with LBP [46,58,59].

We, therefore, conducted a study that aimed to develop a multivariate prognostic model for back pain, leg pain and activity limitation in patients with LBP based on a comprehensive range of commonly used prognostic factors reflective of the biopsychosocial model of health [60]. Following univariate analyses of a range of variables from 300 participants in the STOPS trial, 58 variables progressed to multivariate analysis (Table A1). Five indicators of positive outcome (belonging to either the reducible discogenic pain or disc herniation with associated radiculopathy subgroups, below waist paraesthesia, walking as an easing factor and low transversus abdominis tone) and 10 indicators of negative outcome (both parents born overseas, deep leg symptoms, higher sick leave duration on the Örebro Musculoskeletal Pain Questionnaire [61], high multifidus tone, clinically determined inflammation [62,63], higher back and leg pain severity, lower Oswestry Disability Index [64] lifting capacity, lower capacity for light work (Örebro item) and higher Pain Drawing [65] scores based on percentage body chart coverage) were identified (Table 2).

Researchers and clinical practice guidelines [66] have suggested that biomedical factors are less relevant in the management of non-specific LBP and few studies have identified biomedical or physical factors of prognostic value [67]. However biomedical factors are commonly used by clinicians in decision making [68]. In our study, nine of the 15 prognostic factors related primarily to pathoanatomical mechanisms. In addition, previously reported psychosocial predictors such as depression, fear avoidance and recovery expectations were not prognostic when analysed in a multi-variate model of a comprehensive range of prognostic factors. These results provide support for the validity of the STOPS approach of individualised treatment based on a range of biomedical, psychological and social factors.

### 3.2. Development of an Individualised Physiotherapy Treatment Program

**Figure 1:**
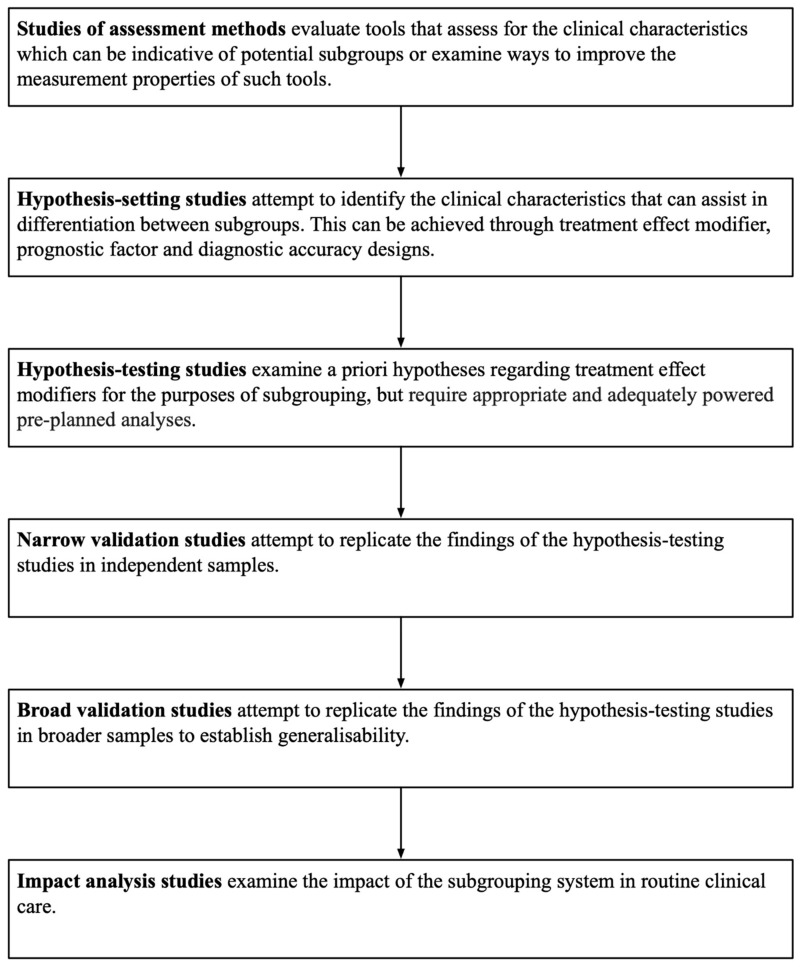
Conceptual phases of subgrouping research (adapted from Kent et al [19]).

Identifying subgroups of different types of LBP is one way of individualising physiotherapy, and treatment targeting specific features or causal mechanisms underpinning the nature of the subgroup has the potential of being more effective in RCTs [13,16]. However, developing a LBP subgrouping system is challenging and the review literature shows that a wide array of approaches exist [13,19,20,21,25,26,27,29,69,70,71,72]. Historically, subgrouping systems have been developed by experts combining the best available evidence with their own clinical experience [73,74,75,76,77]. More recently, a standardised approach to subgroup development used in the medical domain [78] has been extrapolated to LBP [19,79] involving: initial evaluation of assessment methods of potential utility for subgrouping, hypothesis setting studies using a range of methodologies, a priori hypothesis testing studies and a series of further validation stages including analysis of impact of the subgrouping system on routine care (Figure 1). A key component of the hypothesis generation, hypothesis testing and subgroup validation studies is evaluation of treatment effect modifiers within a RCT. Treatment effect modifier studies aim to assess whether the effect of a treatment (relative to a comparison treatment) is different in people with certain characteristics (which are of potential use in defining a subgroup), compared to those without [80]. However, to be adequately powered treatment effect modifiers studies need to be around four times larger than studies investigating overall treatment effect [81,82]. Given the complexity of LBP, a relatively large number of variables require exploration for relevance to subgrouping, which further increases the necessary sample size in treatment effect modifier studies [83]. These issues mean that treatment effect modifier RCTs, with the associated high costs, are of questionable feasibility, particularly in certain research funding contexts such as Australia [71,84,85].

A range of methodologies other than treatment effect modifiers can be used in the hypothesis setting stage of subgroup development, although each approach has significant limitations. Studies evaluating the diagnostic accuracy of different subgroup features/clinical measures are limited by the absence of suitable reference standards [7]. Commonly used reference standards such as imaging, discography and diagnostic blocks have all demonstrated significant false positives due, at least in part, to the complexity of LBP including psychosocial and neurophysiological influences [86]. Despite growing popularity [87] and defined methodological rigour, ‘data driven’ analyses for identifying and developing subgroups also have significant limitations. Statistical processes can result in artificial subgroups [88] of limited clinical use and/or meaningfulness [20,21,83] and a degree of judgement is required in undertaking the analyses with the potential for bias [20,88,89,90].

Contemporary methodologies for the development and validation of subgrouping systems work well in certain medical contexts to allow greater individualisation of treatment [78]. Yet, as described above, extrapolation of these principles to the complex domain of LBP has limited feasibility and methodological shortcomings. An alternative approach is the principle of “convergence of validity” described as when “…evidence supporting or refuting the (subgrouping) system (is) gathered from different sources and from the use of different methods. In the best case scenario, these sources converge and indicate similar meanings of the underlying constructs being studied.” [91] (p. 312). Implicit in this approach is an acceptance of the limitations of all research designs in relation to subgroup development.

As an alternative subgrouping strategy, convergence of validity is consistent with the original definitions of evidence-based practice that emphasise the constructive interaction between the research literature and clinical perspectives [92]. It also aligns with expert recommendations from the field of epidemiology [93] and mirrors the approach taken in other complex medical domains such as the classification of headache [94] and non-Hodgkin’s lymphoma [95].

In essence, a convergence of validity approach is the equivalent to the hypothesis setting phase where a range of research methodologies are considered in developing a subgrouping system. In applying a convergence of validity approach, it is accepted that the complete validation of such a system, particularly through repeated treatment effect modifier studies, is not likely to be feasible. Yet this limitation in achieving full validation should not prohibit the use of the subgrouping system in other research designs, such as RCTs, provided the limitations of system validity are acknowledged.

Four papers [83,96,97,98] have been published in relation to the STOPS trial justifying and outlining detailed individualised treatment protocols on the basis of convergence of validity supporting five subgroups. This process has been further supported by two expert panels [99,100] and five systematic reviews [20,101,102,103,104]. Four of the subgroups were primarily based on clinical features indicative of a pathoanatomical diagnosis of the LBP and comprised: reducible discogenic pain, zygapophyseal joint pain, non-reducible discogenic pain, and disc herniation with associated radiculopathy. A fifth subgroup (multi-factorial persistent pain) captured people without a clear pathoanatomical classification along with likely psychosocial contributors to their delayed recovery as measured on the Örebro Musculoskeletal Pain Questionnaire [61].

Participants with reducible discogenic pain were prescribed a home program based on mechanical loading strategies that led to improvement or centralisation of symptoms. This included repeated/sustained movement exercises, a walking program, taping and postural advice [83]. Participants with zygapophysial joint dysfunction received targeted manual therapy comprising unilateral mobilisation ± manipulation applied with a rigorous clinical reasoning approach [96]. All participants apart from those in the MFP group received motor-control training targeting local muscles such as transversus abdominus leading into a pain contingent graded functional exercise program [96]. This was the primary treatment for participants with disc herniation with associated radiculopathy or non-reducible discogenic pain. Those with multifactorial persistent pain received physiotherapy focusing on psychosocial and neurophysiological rather than pathoanatomical mechanisms [98]. Progression of functional exercise in this subgroup was time-contingent, and cognitive restructuring/behavioural strategies were used targeting key barriers identified on the Örebro Musculoskeletal Pain Questionnaire.

Although subgroup membership determined the primary treatment approach, a range of other treatment components were also provided depending on identification of other pathoanatomical, psychosocial or neurophysiological barriers to recovery. All participants receiving individualised physiotherapy engaged in an explanation/discussion regarding: the nature/source of their symptoms, treatment options available outside of the RCT and timeframes for recovery. Participants also worked with the trial physiotherapists on goal setting, cognitive restructuring of counterproductive beliefs, behavioural strategies to support and reinforce the education program, as well as modifying unproductive behaviours and discharge planning. A range of optional treatment components were provided including: pain management strategies (pharmacological and non-pharmacological), management of inflammation in participants with a clinically determined inflammatory component to their pain, management of work issues, sleep strategies, relaxation and dealing with increases in pain (flare-ups). In participants failing to improve with a pathoanatomical approach initially, the trial physiotherapist determined whether transfer to the MFP treatment protocol was required. These treatment strategies were all applied in a manner individualised to the participant’s presentation as determined by the trial physiotherapist.

### 3.3. Effectiveness of Individualised Physiotherapy

Based on the above-described research, the STOPS trial aimed to evaluate the effectiveness of individualised physiotherapy compared to guideline-based advice. Advice regarding prognosis and resuming normal activities is recommended in all clinical guidelines for people with LBP of over 6-weeks duration [8]. Prior to our clinical trials, there had been few published RCTs evaluating the effectiveness of individualised physiotherapy compared to guideline-based advice.

Other recent subgrouping approaches based on risk stratification such as STarT Back [33] and physical examination findings (i.e., movement patterns) do not address pathoanatomical factors despite this approach being common in clinical practice and the convergence of evidence that it may be important in clinical decision making for LBP [16,83,96,97,98].

Using the STOPS individualised physiotherapy protocol including manual therapy, directional preference management, postural re-education, motor control training, and graded functional exercise [83,96,97,98] we evaluated the effectiveness of individualised physiotherapy compared to guideline-based advice for 300 participants with early persistent LBP (6-weeks to 6-months duration) [43].

**Figure 2:**
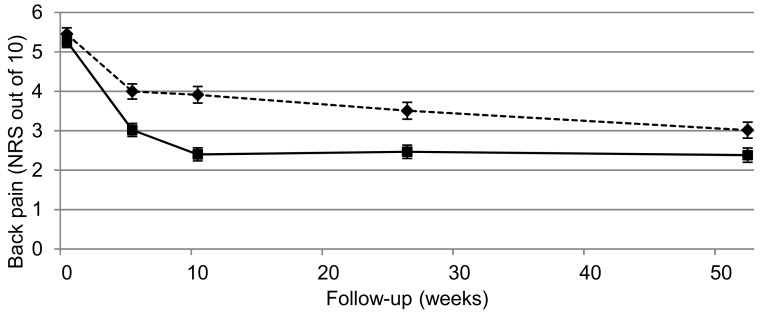

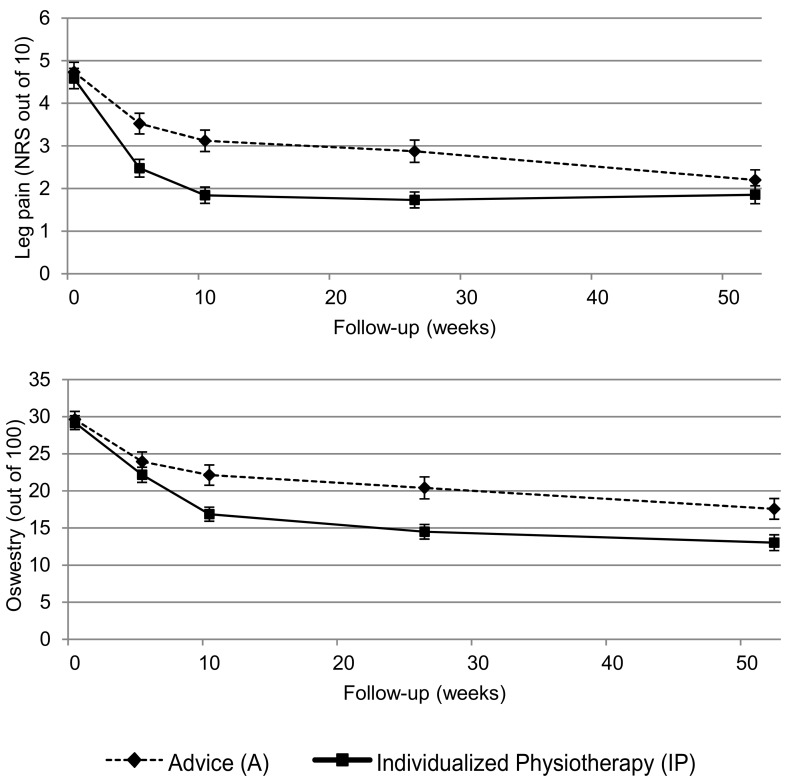
Group mean scores (error bars indicate standard errors) for primary outcomes at baseline and 5-, 10-, 26- and 52-week follow-up in the STOPS Trial (adapted from Ford et al. [43], permission admitted).

Results (Figure 2) showed that individualised physiotherapy was more effective than advice in improving activity limitation (at 10, 26 and 52-weeks) as well as back pain and leg pain (at 5, 10 and 26-weeks). Between-group mean differences were statistically significant in 71% of the primary and secondary outcomes measured in the trial. Participants receiving individualised physiotherapy took 5-8 weeks to achieve the same pain rating as those receiving advice at 12 months indicating a more rapid rate of recovery. Satisfaction with individualised treatment was high, and 92.3% of individualised physiotherapy participants completed the intervention. Based on contemporary definitions, these results are clinically important [105,106].

### 3.4. Cost-Effectiveness of Individualised Physiotherapy

Direct healthcare costs attributable to people with LBP in Western countries seeking healthcare is estimated at billions of dollars annually [107,108] and is predicted to rise [109,110]. Treatments that improve clinical outcomes such as pain and activity at a sustainable cost are urgently needed [111].

Guideline-based advice is a low-cost treatment that is commonly prescribed by medical practitioners [112] and physiotherapists [113]. However, low cost does not necessarily correspond to cost-effectiveness when treatment effects and all relevant costs (healthcare and other) are considered. There is insufficient evidence regarding the cost-effectiveness of advice for LBP according to one systematic review [111].

The STOPS trial showed that individualised physiotherapy was clinically more effective than guideline-based advice [43]. Given the treatment cost of delivering individualised physiotherapy (10 sessions) was higher than advice (2 sessions), consideration is required as to whether the larger effects were worth the additional cost. We therefore investigated the cost-effectiveness of individualised physiotherapy versus advice in people with LBP enrolled in the STOPS trial [114].

The results showed that total health care costs were similar for both groups despite individualised physiotherapy being more expensive than guideline-based advice (Table 2). This was due to 61% of participants receiving advice seeking further non-medical treatment outside the trial compared to 39% of participants receiving individualised physiotherapy (Table 2). Health benefits favoured individualised physiotherapy over advice (incremental Quality Adjusted Life Years = 0.06 (95%CI: 0.02 to 0.10)). Cost-effectiveness was established by the achievement of an Incremental Cost Effectiveness ratio of $US 422 per quality adjusted life year gained [114]. In addition, lower work absence across the 12-month follow-up resulted in income savings of $US 1995 (95%CI: 144 to 3847) per working participant in the individualised physiotherapy group compared to the advice group (Table 2).

### 3.5. Who Benefits Most from Individualised Physiotherapy Versus Advice?

Treatment effect modifier studies are helpful for determining characteristics of patients who respond best to a particular treatment relative to another in an RCT [19]. The STOPS treatment effect modifier study investigated several patient characteristics identified a priori and listed on the trial register [115] based on the hypothesis that participants with more severe, persistent or complex LBP would derive the largest benefits from individualised physiotherapy relative to advice. This hypothesis was supported by the results showing that participants with higher back pain intensity, higher Örebro scores (indicative of higher risk of persistent pain) or longer duration of symptoms derived the largest benefits from individualised physiotherapy relative to advice. These findings are of particular importance because the presence of these characteristics has been associated with a worse prognosis as well as higher treatment and societal costs [107,116,117]. Targeting individualised physiotherapy towards these higher risk groups may, therefore, result in even stronger treatment effectiveness and cost-effectiveness than those reported for the whole sample involved in the STOPS trial.

## 4. Discussion

Research into individualised physiotherapy is a high research priority that has, to date, yielded disappointing results in RCTs. The series of studies described in this paper support the development and validity of the STOPS subgrouping approach. In addition, three studies on the effectiveness, cost-effectiveness and treatment effect modifiers provide further support for the individualised treatment of LBP using the STOPS approach. We are unaware of any similar body of research on the utility of individualised physiotherapy based on a comprehensive biopsychosocial-based treatment model. There are a range of possible factors that may have contributed to the above-described results.

### 4.1. The Definition of Clinical Importance

Most RCTs on LBP demonstrating statistically significant results show small effects of questionable clinical importance [8,9]. However, the traditional definition of clinical importance based on the minimal clinically important difference (MCID) has been questioned given it was developed for use on individuals rather than group data and may not be appropriate for people with lower severity symptoms [105,118]. Authoritative contemporary guidelines recommend determining clinical importance using multiple methods of analysis including consistency of results across multiple primary and secondary outcome measures, risks/benefits of the treatments, consideration of the population being sampled, and the proportion of individual patients who demonstrate change in outcome measures in excess of the MCID *in addition* to between-group differences in mean scores [106,119,120].

The STOPS trial did not demonstrate clinically important between-group mean differences based on the MCID. However, in accordance with our a priori statistical plan [121], a primary outcome responder analysis was conducted. This analysis showed that participants receiving individualised physiotherapy had 1.8 and 1.6 times the chance of improving by at least 50% from baseline on back and leg pain, respectively, at the 10-week follow-up compared with those receiving advice alone. By 52 weeks, those having individualised physiotherapy also had 1.5 times the chance of improving by 50% from baseline on the Oswestry Disability Questionnaire compared with those receiving advice. All secondary outcomes favoured individualised physiotherapy, with the exception of work interference, but the cost-effectiveness study showed significantly lower work absence (and associated lost income) in the individualised physiotherapy group. In the secondary-outcomes responder analysis, participants receiving individualised physiotherapy had 1.3–4.1 times the chance of achieving a clinically important change compared with those receiving advice. Participant satisfaction was significantly greater and non-medical co-interventions significantly lower in the individualised physiotherapy group. All between-group comparisons should be interpreted in the context of large *within-group* improvements on all primary outcomes for both treatment groups [33]. Given the population sampled were ≥6-weeks post-injury where spontaneous recovery is limited [4,5], it is likely that both treatments were helpful, with individualised physiotherapy conferring additional benefits over and above advice. There were no serious adverse events in either group and with detailed published clinical protocols available, the STOPS approach to LBP is potentially accessible worldwide without extensive training common to other individualised physiotherapy approaches [33,122].

The clinical importance of the between-group differences as a measure of significance in the STOPS trial is further strengthened by the cost-effectiveness analysis. Results showed an incremental cost-effectiveness ratio (ICER) of US$422 per quality-adjusted life year (QALY), which compares favourably to other relevant RCTs in the field. Cognitive behavioural therapy is recommended in all clinical guidelines for LBP [8,9], but has an ICER of US$2773 per QALY gained for group cognitive behavioural therapy along with advice versus advice alone [123]. In another relevant RCT [124] five sessions of physiotherapy were not cost-effective compared with one session of advice as there were no significant differences in health outcomes. These data further support the clinical importance of the STOPS trial results by way of cost-effectiveness compared to guideline-based advice.

Another potential indicator of clinical importance from RCTs is the proportion of participants who complete the intervention. High drop-out rates in clinical trials may indicate that the intervention is not acceptable to participants on the basis of ineffectiveness, patient preferences, the required commitment to comply with treatment, or side-effects. Drop-out rates exceeding 15% have been reported for multiple LBP trials of graded activity [125], anticonvulsants [126], and in one trial of individualised physiotherapy [122]. The individualised physiotherapy group in the STOPS trial had a 7.7% drop-out rate, suggesting that the treatment was acceptable for most participants and giving further support to the clinical importance of the STOPS trial results.

### 4.2. Comparison Group Selection and Advice

When designing and interpreting RCTs, it is important for the researcher and consumer to carefully consider the comparison group. Common trial designs in LBP use no treatment, placebo treatment, advice, usual medical care or various types of physiotherapy interventions. There is no right or wrong approach to designing a comparison group in an RCT; it simply informs the hypothesis being tested. For example, a RCT comparing manual therapy to placebo manual therapy is designed to test the specific effect of manual therapy independent of any non-specific effects such as patient expectations, learning/conditioning effects and neurophysiological effects [127].

It is worth reflecting on the use of guideline-based advice in designing a RCT. Advice to stay active and reassurance regarding prognosis is recommended as first-line treatment in all clinical guidelines for acute and persistent LBP as described in Table 1 [9]. The evidence directly supporting the effectiveness and cost-effectiveness of guideline-based advice is sparse [111,128,129,130]. Nevertheless, it has been asserted that this treatment is just as effective as more costly and complex treatments [10,11]. Although not commonly used as the sole treatment approach by practitioners in the field [131], guideline-based advice is being advocated strongly as first line treatment ahead of other physiotherapeutic treatments such as manual therapy as well as medical treatments such as NSAIDs [8,9,131,132]. Given the low-quality evidence supporting guideline-based advice, it is possible that this approach is counterproductive, particularly if second-line treatments are more effective without being prohibitively more expensive. On this basis, guideline-based advice is an important comparison group in a RCT evaluating the effectiveness of commonly used second-line treatments (Table 1).

### 4.3. Use of a Pathoanatomical Approach

A major difference between the STOPS trial and the majority of the LBP research was the incorporation of pathoanatomical factors into the subgrouping approach and pathoanatomical-based decision making in the clinical protocol. Definitive criteria for pathoanatomical-based diagnosis and/or clinical decision making in LBP are not available [8]. It has been suggested that further research into and clinicians hypothesising about pathoanatomical barriers to recovery is likely to be at best, futile and, at worst, counterproductive to patient outcomes [66,133,134]. However, there is sparse evidence supporting this contention [16] and a pathoanatomical approach is common in clinical practice [68]. In addition, although there are likely to be benefits from addressing exercise/activity, lifestyle and psychosocial factors, the prognostic and treatment effects are small [134]. On this basis, it does not seem sensible to abandon clinical and research-based hypothesising on the role of pathoanatomy unless compelling evidence to do so is provided. Guideline-based advice is not informed by pathoanatomy and the mechanisms of effect are likely to be non-specific. As such, it is possible that a reason for the significant between-group differences in the STOPS trial was that assessment and treatment incorporated hypothesised pathoanatomical diagnoses and related clinical decision making. This premise was supported by the prevalence of pathoanatomical factors in the STOPS prognostic study and the convergence of results from a range of other research designs [83,96,97,98] 

One example of this approach is the STOPS protocol, where treatment was individualised based on the hypothesised presence or absence of an inflammatory component to the LBP. The lumbar intervertebral disc is a biologically plausible contributor to LBP [135]. The mechanisms underpinning the symptoms of disc related pain and activity limitation are unclear, however substantial evidence exists supporting the role of inflammation in disc degeneration and disc herniation with associated radiculopathy (DHR) [136,137]. Studies investigating the composition and structure of lumbar discs have shown fibrosis, vascular invasion, inflammatory granulation tissue formation and extensive innervation along fissures in the posterior annulus fibrosis in painful degenerative discs and around symptomatic nerve roots. Such changes are not observed in non-painful degenerative or herniated discs [138,139].

Further evidence on the presence of and potential importance of inflammatory processes in degenerative discs and DHR [140] can be seen in disc tissue histologically [141,142,143,144,145], in the disc tissue using other inflammatory markers [146,147,148,149,150,151] and as measured by serum biomarkers in people with LBP [152]. A recent study showed that high serum tumour necrosis factor in acute LBP predicted poor recovery of pain and activity limitation at 6-months, providing further evidence of the relevance of inflammation [153].

Significant evidence suggests that inflammatory processes are a potential treatment target in clinical trials [136,137,154,155,156], particularly in people who may have discogenic pain [141,142,143,144,145]. Clinical features of inflammatory back pain such as spondyloarthropathy have been validated using practitioner surveys, expert panels and diagnostic accuracy studies [63,157,158,159,160]. These features include age <40 years, insidious onset, improvement with exercise, no improvement with rest and pain at night with improvement upon getting up from bed [161]. These results are similar to studies on the clinical features of disc related LBP and associated inflammation [144,145,147].

Although systematic reviews and guidelines suggest that NSAIDs are only a second-line treatment option for the management of LBP (Table 1), the literature above suggests that management of inflammatory processes might be more effective if targeted to individuals with clinical symptoms indicative of inflammation, particularly with a combination of pharmacological and other relevant management strategies. The clinical features suggestive of the presence of inflammation informed clinical decision making in the STOPS trial regarding when to implement anti-inflammatory treatment such as medication, taping, postural management and gentle walking. The identification of an inflammatory component was also important where inflammation may have hindered the effectiveness of mechanically based treatment approaches such as exercise, manual therapy or directional preference management [162,163]. The STOPS trial was unique in identifying clinically determined inflammation as a reason for exercising caution with mechanical treatment and simultaneously, treating inflammatory problems using anti-inflammatory treatment. The guideline-based advice comparison treatment gave no consideration to the role of inflammation. Therefore, clinical decision making based on the possible presence/absence of inflammation may have been a factor contributing to the significant between-group differences. This premise is further supported by the significance of clinically determined inflammation identified in the STOPS prognosis study.

### 4.4. Treatment Fidelity in Randomised Controlled Trials

Methods to maximise treatment fidelity in RCTs for physiotherapy interventions are highly variable and often poorly reported [164,165,166,167,168,169]. The STOPS trial employed a range of evidence-based methods [170] to enhance treatment fidelity including: specification regarding the treatment program design (140 page clinical manual with full detail on all aspects of individualised treatment); 16 hours of standardised practitioner training; review of practitioner treatment and practitioner feedback during the RCT (by way of study researchers reviewing the clinical notes followed by verbal feedback and group-based monthly case reviews); and evaluation of the participant’s perspective/understanding of the treatment provided (qualitative exit interviews). Participants also completed exercise diaries that were checked by the physiotherapist at each visit. Similar methods were put in place with the advice treatment program. Given the relative complexity of the individualised physiotherapy, it is plausible that the treatment fidelity program would have had greater impact on patient outcomes in the individualised physiotherapy group. This could, therefore, have been an additional factor for the significant between-group differences observed in the STOPS trial.

### 4.5. The Importance of Motor Control

Motor control retraining focusing on posture, movement and muscle activation was a significant component of individualised physiotherapy for all participants apart from those in the multifactorial persistent pain (MFP) subgroup. The relevance and effectiveness of this approach is contentious [171] and there are significant inconsistencies in systematic review results [172,173,174]. Nevertheless, as the advice group did not receive motor control training this treatment component could have been responsible in part for the significant between-group effects. This premise is supported by the significance of suboptimal motor control identified in the STOPS prognosis study as well as the biological plausibility and potential pathoanatomical relevance of optimising motor control in people with LBP [175,176,177,178].

## 5. Clinical Implications

The clinical implications of the research presented in this paper are potentially substantial but need to be contextualised within an evidence-based framework. In order to make strong recommendations to practitioners, the findings need to be replicated in independent samples and/or systematic reviews updated to incorporate the relevant data into meta-analyses. However, the significance and consistency of the results are sufficient to challenge some of the common perceptions around evidence-based practice for LBP.

Guidelines routinely state that the vast majority of LBP patients should be considered as a non-specific condition where consideration of pathoanatomy is not possible or necessary [8,9]. Some guidelines go so far as to state that clinical decision making based on pathoanatomy may be harmful [66] despite sparse data to support this assertion. The results presented in this paper support the notion that hypothesising on and clinical decision making with regard to pathoanatomical considerations cannot be discounted and may lead to superior outcomes compared to a less targeted approach.

There is sparse data supporting the effectiveness of simple guideline-based advice. It is of interest that the RCT with the largest effect sizes in favour of advice incorporated a pathoanatomical explanation [179]. Despite this, advice in the absence of a pathoanatomical explanation is being recommended as first-line treatment for LBP of any duration [8,9]. The series of studies described in this paper suggest that further consideration and evaluation of guideline-based advice as a first-line treatment is required.

The generalisability of the STOPS trial results should be superior to most recent RCTs on individualised physiotherapy, where only a few experienced practitioners were used [122] and/or detailed clinical protocols were not published [33,122]. The treatment used in the STOPS trial was provided by physiotherapists with a range of experience, none of whom had a post-graduate qualification. It encompassed the most commonly used methods by physiotherapists [113,180,181] however the published treatment protocols have the potential to improve the quality of existing standards in clinical practice due to the detailed explanations and clinical decision making processes provided.

On the basis of the STOPS trial, practitioners can provide their patients with an average timeframe for expected treatment outcomes when receiving individualised physiotherapy. Patients are likely to experience rapid reductions in back/leg pain in the first 10 weeks of treatment, but optimal improvements in activity limitation are likely to take longer. Patients will also be reassured regarding the cost-effectiveness of the treatment, particularly with regards to minimising time off work.

## 6. Future Research

The results of this series of studies should be highly impactful on future research. Much of the research in LBP develop study designs, eligibility criteria, prognostic factors or treatment protocols that are relatively simplistic in nature. Whilst this approach renders research projects more feasible and potentially more methodologically rigorous, it does not reflect the real-world complexity of LBP and the associated treatment options that are likely to be most effective.

A pathoanatomical approach should be considered when planning future clinical research within the context of a truly biopsychosocial model for LBP.

More research is required on the relative importance of different components of individualised physiotherapy. Individualised physiotherapy also needs to be compared to other comparison groups and on different populations, particularly persistent LBP where more entrenched psychosocial and neurophysiological barriers to recovery are likely to be relevant.

Researchers should be encouraged by the clinical importance of the results in the STOPS trial and be emboldened to develop ambitious research hypotheses based on an in-depth understanding of both clinical and research perspectives.

Greater rigour should be applied to the development of clinical guidelines to ensure that low-quality evidence such as the sparse data and questionable cost-effectiveness supporting simple advice is acknowledged.

Researchers should follow the lead of the STOPS trial in providing detailed clinical protocols that are feely available in full-text. Such an approach would greatly accelerate the dissemination of evidence-based information to practitioners in the field and substantially improve external validity.

## 7. Conclusions

LBP is the most burdensome health problem in the world. Prior to the publication of the studies in this body of research, there was limited evidence for the effectiveness of individualised physiotherapy and a focus in clinical guidelines on advice as first-line treatment for LBP. Our series of studies challenge the role of advice alone in early persistent pain and suggests that the concept of non-specific LBP needs to be reconsidered. Furthermore, there are now detailed clinical protocols and quality evidence to support the STOPS approach to individualised physiotherapy in clinical practice and future research studies.

## Figures and Tables

**Table 1:** Overview of interventions endorsed for non-specific low-back pain in evidence-based clinical practice guidelines (adapted from Foster et al. 2018) [9].

	Acute LBP (<6 weeks)	Persistent LBP (>12 weeks)
First line care	Advice Education	Advice Education Exercise CBT
Second line or adjunctive care	NSAIDs Superficial heat Manual therapy Massage Acupuncture	NSAIDs Selective norepinephrine reuptake inhibitors Manual therapy Acupuncture Yoga Mindfulness Interdisciplinary rehabilitation Discectomy or laminectomy for disc herniation with associated radiculopathy
Limited use in selected patients	Opioids Skeletal muscle relaxants Exercise CBT	Opioids Epidural injection
Not recommended	Paracetamol Systemic glucocorticoids Epidural injection	Paracetamol Systemic glucocorticoids
Insufficient evidence	Mindfulness Interdisciplinary rehabilitation Selective norepinephrine reuptake inhibitors Antiseizure medication Any surgery	Superficial heat Skeletal muscle relaxants

CBT = cognitive behavioural therapy, NSAIDs = non-steroidal anti-inflammatory drugs.

**Table 2:** Back related healthcare utilization and costs per patient.

Resource	Resource Use: Units/Patient (SD), % of Patients Utilizing		Cost/Patient (SD) in US$		
	IP	Advice	IP	Advice	Between-Group Cost Difference (95% CI) *
Study physiotherapy	8.9 (2.1), 100%	1.8 (2.4), 99%	379.35 (87.10)	81.93 (18.46)	**297.72 (282.85 to 312.01)**
Medical consultations	1.7 (5.3), 32%	2.0 (4.2), 40%	86.95 (280.78)	110.55 (238.03)	−23.61 (−85.61 to 38.40)
Medical intervention Surgery (discectomy) Injections	0.01 (0.08), 0.7% 0.1 (0.3), 3.4%	0.02 (0.12), 1.5% 0.1 (0.5), 7.7	35.81 (434.18) 5.87 (36.32)	80.99 (650.40) 13.82 (56.10)	−45.18 (−174.68 to 84.32) −7.95 (−19.01 to 3.10)
Allied health consultations	3.3 (6.3), 38.8%	7.9 (12.3), 60.8%	152.38 (292.15)	324.47 (480.14)	**−172.09 (−264.94 to −79.25)**
Medication	57.0%	54.6%	59.87 (140.54)	85.60 (207.93)	−25.73 (−69.16 to 17.69)
Total Healthcare cost (95%CI)			782.82 (623.82 to 941.82)	755.79 (592.84 to 918.75)	27.03 (−200.29 to 254.35)
Work absence: Mean (95%CI), %	10.8 (4.6 to 17.1) days, 36%	20.5 (13.3 to 27.6) days, 44%	$1889.16 (680.86 to 3097.46)	$3884.67 (2497.22 to 5272.12)	**$ −1995.51 (−3847.03 to −143.98)**

IP = individualised physiotherapy; SD = standard deviation; *, Between-group comparisons analysed via linear mixed models, with positive values representing a higher cost in the individualised physiotherapy group relative to the advice group, significant between-group differences in bold.

## Appendix A

**Table A1:** Prognostic factors for Oswestry, back pain and leg pain obtained from the multivariate model.

	Oswestry (0–100)			Back Pain (0–10)			Leg Pain (0–10)		
Prognostic factor	*N*	B Coefficient (95% CI)	*p*-value	*N*	B Coefficient (95% CI)	*p*-value	*N*	B Coefficient (95% CI)	*p*-value
Intercept		−0.2 (−14.4 to 13.9)	0.975		0.0 (−1.962 to 2.019)	0.977		−2.0 (−4.3 to 0.4)	0.095
Subgroup Disc herniation/radiculopathy *	54	−1.3 (−5.0 to 2.3)	0.473 +	54	**−0.6 (−1.2 to −0.1)**	**0.029 +**	54	−0.3 (−0.9 to 0.3)	0.312 +
Reducible discogenic pain *	78	−3.2 (−5.9 to −0.6)	0.017 +	78	**−0.9 (−1.3 to −0.4)**	**<0.001 +**	70	**−0.7 (−1.2 to −0.2)**	**0.005 +**
Manual therapy group *	64	−2.6 (−5.5 to 0.3)	0.082 +	64	−0.5 (−1.0 to 0.0)	0.050 +	49	−0.2 (−0.7 to 0.4)	0.560 +
Multifactorial persistent pain *	8	−1.3 (−7.8 to 5.1)	0.683 +	8	0.2 (−0.5 to 0.8)	0.647	7	−0.2 (−1.4 to 0.9)	0.725 +
Parents born overseas Both born overseas #	165	**3.4 (1.1 to 5.7)**	**0.004**	165	**0.6 (0.2 to 0.9)**	**0.003**	141	**0.5 (0.1 to 0.9)**	**0.026**
One born overseas #	21	0.7 (−3.7 to 5.1)	0.761	21	0.2 (−0.4 to 0.8)	0.570	18	0.1 (−0.5 to 0.8)	0.723
Paresthesia below waist	134	**−3.3 (−6.0 to −0.6)**	**0.016 +**	134	−0.1 (−0.5 to 0.3)	0.607 +	125	−0.2 (−0.6 to 0.3)	0.498 +
Deep leg symptoms	145	2.5 (0.0 to 4.9)	0.053	145	0.2 (−0.2 to 0.6)	0.286	145	**0.6 (0.2 to 1.0)**	**0.002**
Walking eases symptoms	160	−2.0 (−4.2 to 0.2)	0.073 +	160	**−0.5 (−0.9 to −0.2)**	**0.005 +**	138	−0.2 (−0.6 to 0.2)	0.273 +
Lateral flexion limited by pain	116	1.0 (−1.3 to 3.4)	0.382	116	0.1 (−0.3 to 0.5)	0.536	106	0.4 (0.0 to 0.8)	0.055
Transversus abdominis low tone	109	**−3.0 (−5.3 to −0.7)**	**0.012 +**	109	**−0.8 (−1.1 to −0.4)**	**<0.001 +**	91	−0.4 (−0.9 to 0.0)	0.051
Multifidus high tone	60	2.0 (−1.2 to 5.1)	0.215	60	0.0 (−0.4 to 0.5)	0.918	47	**0.7 (0.1 to 1.4)**	**0.019**
Clinical inflammation	182	1.1 (−1.1 to 3.3)	0.342	182	**0.4 (0.1 to 0.8)**	**0.020**	165	−0.2 (−0.6 to −0.2)	0.311 +
Back pain severity	300	0.2 (−0.5 to 0.8)	0.556	300	**0.3 (0.2 to 0.4)**	**<0.001**	261	0.1 (0.0 to 0.2)	0.245
Leg pain severity	300	0.1 (−0.5 to 0.6)	0.764	300	0.0 (−0.1 to 0.1)	0.896	261	**0.3 (0.2 to 0.4)**	**<0.001**
Örebro sick leave duration (0–10)	300	**1.1 (0.4 to 1.7)**	**0.002**	300	0.1 (0.0 to 0.2)	0.112	261	0.1 (0.0 to 0.2)	0.219

*, relative to “non-reducible discogenic pain”’ #, relative to “both parents born in Australia”; +, Positive prognostic indicator. Results are independent of time point, significant p-values in bold. Negative B-coefficients represent lower outcome scores and therefore a better outcome at follow-up in participants with the listed prognostic factor. Predicted outcome for a given patient can be calculated by applying the patient’s score on each baseline factor to the B-coefficients, and adding the scores from each item together (including the intercept).
